# Analysis of meniscal degeneration and meniscal gene expression

**DOI:** 10.1186/1471-2474-11-19

**Published:** 2010-01-28

**Authors:** Yubo Sun, David R Mauerhan, Patrick R Honeycutt, Jeffrey S Kneisl, James H Norton, Edward N Hanley, Helen E Gruber

**Affiliations:** 1 Department of Orthopaedic Surgery, Carolinas Medical Center, PO Box 32861, Charlotte, NC 28232, USA; 2 Department of Biostatistics, Carolinas Medical Center, PO Box 32861, Charlotte, NC 28232, USA

## Abstract

**Background:**

Menisci play a vital role in load transmission, shock absorption and joint stability. There is increasing evidence suggesting that OA menisci may not merely be bystanders in the disease process of OA. This study sought: 1) to determine the prevalence of meniscal degeneration in OA patients, and 2) to examine gene expression in OA meniscal cells compared to normal meniscal cells.

**Methods:**

Studies were approved by our human subjects Institutional Review Board. Menisci and articular cartilage were collected during joint replacement surgery for OA patients and lower limb amputation surgery for osteosarcoma patients (normal control specimens), and graded. Meniscal cells were prepared from these meniscal tissues and expanded in monolayer culture. Differential gene expression in OA meniscal cells and normal meniscal cells was examined using Affymetrix microarray and real time RT-PCR.

**Results:**

The grades of meniscal degeneration correlated with the grades of articular cartilage degeneration (r = 0.672; P < 0.0001). Many of the genes classified in the biological processes of immune response, inflammatory response, biomineral formation and cell proliferation, including major histocompatibility complex, class II, DP alpha 1 (*HLA-DPA1*), integrin, beta 2 (*ITGB2*), ectonucleotide pyrophosphatase/phosphodiesterase 1 (*ENPP1*), ankylosis, progressive homolog (*ANKH*) and fibroblast growth factor 7 (*FGF7*), were expressed at significantly higher levels in OA meniscal cells compared to normal meniscal cells. Importantly, many of the genes that have been shown to be differentially expressed in other OA cell types/tissues, including ADAM metallopeptidase with thrombospondin type 1 motif 5 (*ADAMTS5*) and prostaglandin E synthase (*PTGES*), were found to be expressed at significantly higher levels in OA meniscal cells. This consistency suggests that many of the genes detected in our study are disease-specific.

**Conclusion:**

Our findings suggest that OA is a whole joint disease. Meniscal cells may play an active role in the development of OA. Investigation of the gene expression profiles of OA meniscal cells may reveal new therapeutic targets for OA therapy and also may uncover novel disease markers for early diagnosis of OA.

## Background

Osteoarthritis (OA) is a disease characterized by the breakdown of articular cartilage and the formation of osteophytes. However, it has been gradually realized that OA is not merely an articular cartilage disease, but a disease of the whole joint [1,2]. OA synovial membrane and subchondral bone have drawn considerably attention recently. Aberrant gene expression in OA synovium, OA fibroblast-like synoviocytes (FLS) and OA subchondral bone has been detected [3-6]. These findings suggest that OA synoviocytes and subchondral bone cells may be involved in the disease process of OA.

Few studies have investigated the potential role of OA meniscal cells in the disease process of OA. The knee menisci are specialized tissues that play a vital role in load transmission, shock absorption and joint stability. Knee menisci may absorb most of the shock generated at the joint because their combined mass is greater than that of the articular cartilage. The current dogma is that menisci protect the articular cartilage, but play a minimal role in the disease process of OA unless they are injured. However, increasing evidence suggests that knee menisci may not be passive bystanders in the disease process of OA. It has been reported that meniscal degeneration is a feature of OA knee joints as revealed by magnetic resonance imaging [7,8]. There is a strong association between meniscal damage/degenerative tears and cartilage loss [9]. In addition, it has been found that meniscal degeneration contributes to joint space narrowing [10]. Taken together, these findings and observations suggest that pathological changes and extracellular matrix degeneration also occur in OA menisci. OA menisci, similar to OA synovial membrane and OA subchondral bone, may play an active role in the disease process of OA.

In the present study, we examined the prevalence of meniscal degeneration in OA patients who underwent joint replacement surgery, and analyzed the correlation between the degeneration of menisci and the degeneration of articular cartilage. We also examined differential gene expression between OA meniscal cells and normal meniscal cells to test the hypothesis that OA meniscal cells are different from normal meniscal cells and may display a disease-specific gene expression profile. The determination of the differential gene expression between OA and normal meniscal cells may not only provide experimental evident to support our hypothesis, but also may reveal new therapeutic targets for OA therapy and uncover novel disease markers for early diagnosis of OA.

## Methods

### Menisci and articular cartilage specimens

Menisci and articular cartilage specimens were collected from OA patients who underwent joint replacement surgery and from osteosarcoma patients who underwent lower limb amputation surgery with the approval of the authors' Institutional Review Board. The need for informed consent was waived since the menisci and articular cartilage were surgical waste of routine joint replacement surgery and lower limb amputation surgery, and there was no patient private information being collected. Specimens were transported to the laboratory from the operation room at our hospital in sterile tissue culture medium. The medial menisci and the medial articular cartilage of the tibia were graded (by DRM, PRH and YS) according to following scales. For the medial meniscus: 0 = normal appearing surface; 1 = minimal fibrillation and degeneration; 2 = moderate fibrillation and degeneration; 3 = severe fibrillation and degeneration, no tears; 4 = severe fibrillation and degeneration, multiple incomplete tears, or complete tears. For medial articular cartilage of the tibia: 0 = normal-appearing surface; 1 = minimal fibrillation and degeneration; 2 = erosion extending to middle layers; 3 = erosion extending into the deep layers; 4 = erosion extending to the subchondral bone, and 5 = the majority of articular cartilage completely absent.

### Preparation and culture of meniscal cells

Dulbecco's modified eagle medium, fetal bovine serum, stock antibiotic and antimycotic mixture were products of Invitrogen (Carlsbad, CA). Meniscal cells were isolated from meniscal specimens as previously described [11]. Briefly, when meniscal specimens were arrived in sterile tissue culture medium, the central region of the medial meniscus was incised, and processed to remove fatty and synovial tissues. These meniscal specimens were then minced into small pieces (3 mm × 3 mm), and cultured in 100 mm plates at 37°C in medium containing 0.5% antibiotic/antimycotic solution and 10% serum. Every three days, medium was changed. Meniscal cells proliferated, migrated out the tissues, attached to the plate and expanded. When meniscal cells reached 80% confluence, they were replated and expanded in monolayer culture. Passage two cells were used in all studies. Meniscal cells prepared from meniscal specimens derived from osteosarcoma patients were used as normal control meniscal cells.

### RNA extraction and microarrays

OA meniscal cells and normal meniscal cells (passage 2) were plated in 100 mm plates at 75% confluence. On the second day, medium containing 1% serum was added and cells were cultured for twenty-four hours. Medium (1% serum) was changed again and cells cultured for another twenty-four hours. Total RNA was extracted using Trizol reagent (Invitrogen, Carlsbad, CA), and purified using Oligotex kit (Qiagen, Valencia, CA). Microarray analyses were performed using these RNA samples. For microarray analysis, double stranded DNA was synthesized from RNA samples using SuperScript double-stranded cDNA synthesis kit (Invitrogen, Carlsbad, CA). The DNA product was purified using GeneChip sample cleanup module (Affymetrix, Santa Clara, CA). cRNA was synthesized and biotin labeled using BioArray high yield RNA transcript labeling kit (Enzo Life Sciences, Farmingdale, NY). The product was purified using GeneChip sample cleanup module and subsequently chemically fragmented. The fragmented, biotinylated cRNA was hybridized to HG-U133_Plus_2 gene chip (Affymetrix, Santa Clara, CA) using Affymetrix Fluidics Station 400. The fluorescent signal was quantified during two scans by Agilent Gene Array Scanner G2500A (Agilent Technologies, Palo Alto, CA), and GeneChip operating Software (Affymetrix, Santa Clara, CA).

### Real-time RT-PCR

cDNA was synthesized using TaqMan^® ^Reverse Transcription Reagents (Applied Biosystems, Inc., University Park, IL). Quantification of relative transcript levels for selected genes and the housekeeping gene glyceraldehyde 3-phosphate dehydrogenase (GAPDH) was performed using the ABI7000 Real Time PCR system (Applied Biosystems, Inc., University Park, IL). TaqMan^® ^Gene Expression assays were used, which contains a FAM-MGB probe for fluorescent detection. cDNA samples were amplified with an initial Taq DNA polymerase activation step at 95°C for 10 minutes, followed by 40 cycles of denaturation at 95°C for 15 seconds and annealing at 60°C for one minute. For each gene, Ct values were obtained in triplicates. Fold change was calculated [12] and the expression level of the genes of interest was normalized to *GAPDH*. Each real time RT-PCR experiment was repeated twice in triplicate.

### Statistical Analysis

The Spearman's correlations between the grades of meniscal degeneration and the grades of articular cartilage degeneration were calculated with SAS^® ^software (version 9.1). A two-tailed p-value of < 0.05 was considered statistically significant. For microarray analysis, Genesifter software (VizX Labs, Seattle, WA) was used to determine fold changes in gene expression, and gene ontologies. Statistical significance was determined using the Student t-test (p < 0.05). A correction factor for false discovery rate was applied using the Benjamini and Hochberg method [13].

## Results

### Degeneration of menisci in OA patients

**Figure 1:**
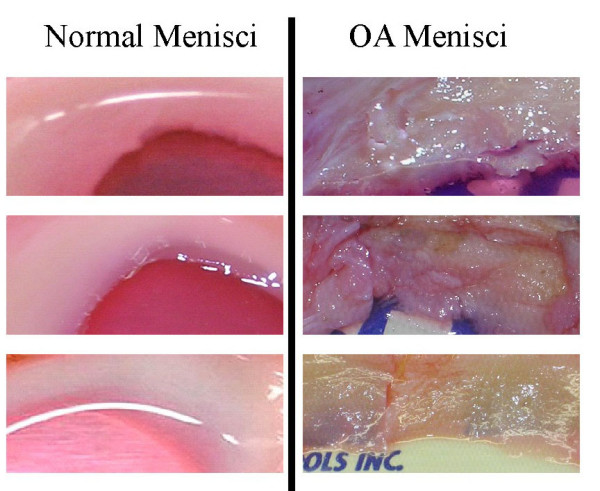
**Representative photos of normal control menisci and OA menisci**. Control menisci (left, top to bottom) were obtained from a 39 year old female osteosarcoma patient, a 43 year old male osteosarcoma patient and a 12 year old female osteosarcoma patient underwent lower limb amputation surgery. OA menisci (right, top to bottom) were obtained from a 65 year old female OA patient, a 67 year old female OA patient and a 50 year old female OA patient.

Demographic patient features and assigned grades are presented in Table 1. All menisci had signs of degeneration. More than 80% menisci showed signs of severe degeneration (grade 4 or grade 3). Representative images of grade 4 OA menisci and grade 0 normal control menisci are shown in Figure 1. Grade 4 OA menisci displayed discolorations and an extremely rough surface. Severe degeneration and fissures/tears were apparent. In contrast, grade 0 control menisci displayed a smooth, white and glistening surface, with no any signs of degeneration.

**Table 1:** Demographic data and grades of articular cartilage and menisci*

I	Cartilage Grade	# Subjects	Mean Age ± SD
	0	1 F	39

	0	1 F	12

	0	1 M	43

	2	3 F, 1 M	62 ± 13

	3	6 F, 5 M	64 ± 9

	4	15 F, 4 M	62 ± 11

	5	12 F, 5 M	67 ± 9

**II**	**Meniscus Grade**	**# Subjects**	**Mean Age ± SD**

	0	1 F	39

	0	1 F	12

	0	1 M	43

	2	4 F, 3 M	57 ± 12

	3	12 F, 6 M	64 ± 9

	4	20 F, 6 M	66 ± 9

*The first three patients are osteosarcoma patients. The rest fifty-one patients are OA patients. F, female; M, male

The relationship between the grade of menisci and the grade of articular cartilage was examined. A correlation between the grade of menisci and the grade of articular cartilage was found. The Spearman's correlation coefficient (r) was 0.672 (p < 0.0001). Of the seventeen OA patients who had grade 5 (the highest grade of articular cartilage degeneration) articular cartilage, fourteen patients (82%) had grade 4 medial meniscus (the highest grade of meniscal degeneration) and three patients had grade 3 medial meniscus. Of the nineteen OA patients who had grade 4 articular cartilages, ten patients had grade 4 (58.82%) and seven had grade 3 (41.17%) medial meniscus. Only two patients had grade 2 (0.11%) medial meniscus.

### Differential gene expression

Microarray analyses of the differential gene expression between OA meniscal cells prepared from menisci (grade 4) derived from five OA patients and normal meniscal cells prepared from menisci (grade 0) derived from three osteosarcoma patients were carried out as described in Methods. The row microarray data can be found in Gene Expression Omnibus (GEO; http://www.ncbi.nlm.nih.gov/projects/geo/) with the accession number #GSE19060. Table 2 presents the demographic data of the meniscal specimens which were the basis of this analysis. Of more than 50,000 transcripts examined, 505 transcripts that displayed differential gene expression (more than 1.2 fold) were identified; 149 transcripts were expressed at significantly higher levels and 356 transcripts at significantly lower levels in OA meniscal cells compared to normal meniscal cells. The differentially expressed genes were classified according to gene ontology category biological process using GeneSifter software (VizX Labs, Seattle, WA). Selected genes, which fell into specific biological processes previously implicated in OA or suspected to play a role in OA, or displayed a distinct expression pattern, are listed in Table 3.

**Table 2:** Demographic data and grades of menisci that were used to prepare meniscal cells*

Patients	Age/Gender/Diagnosis	Grade Meniscus
A	39/F (osteosarcoma)	0

B	43/M (osteosarcoma)	0

C	12/F (osteosarcoma)	0

1	65/F (OA patient)	4

2	56/F (OA patient)	4

3	50/F (OA patient)	4

4	52/F (OA patient)	4

5	61/M (OA patient)	4

* F, female; M, male; OA, osteoarthritis

**Table 3:** Genes differentially expressed in OA meniscal cells compared to normal meniscal cells.

Biological process	Gene Name	Gene ID	Differ Exp (fold)*	Description
Immune response				

	HLA-DPA1	M27487	5.3	Major histocompatibility complex, class II, DP alpha 1

	IFI6	NM_022873	2.0	Interferon, alpha-inducible protein 6

	CFH	X04697	1.7	Complement factor H

	CTSS	NM_004079	1.6	Cathepsin S

	FYN	S74774	1.5	FYN oncogene related to SRC, FGR, YES

	ILF2	NM_004515	-1.3	Interleukin enhancer binding factor 2, 45 kDa

	PSMB9	AI375915	-1.2	Proteasome (prosome, macropain) subunit, beta type, 9

Inflammatory Response				

	ITGB2	NM_000211	6.6	Integrin, beta 2 (complement component 3 receptor 3 and 4 subunit)

	CFH	X04697	1.7	Complement factor H

	BDKRB2	NM_000623	1.3	Bradykinin receptor B2

Cytokine production				

	SRGN	NM_002727	2.8	Serglycin

	ARNT	AI768497	2.3	Aryl hydrocarbon receptor nuclear translocator

	INHBA	M13436	1.9	Inhibin, beta A

Calcium ion transport				

	CACNA1C	F11066	1.5	Calcium channel, voltage-dependent, L type, alpha 1C subunit

	FYN	S74774	1.5	FYN oncogene related to SRC, FGR, YES

	CHRNB2	NM_000748	1.4	Cholinergic receptor, nicotinic, beta 2 (neuronal)

Biomineral formation				

	ENPP1	BF057080	1.7	Ectonucleotide pyrophosphatase/phosphodiesterase 1

	SRGN	NM_002727	2.8	Serglycin

	ACVR2A	AI149508	1.5	Activin A receptor, type IIA

Cell proliferation				

	ITGB2	NM_000211	6.6	Integrin, beta 2 (complement component 3 receptor 3 and 4 subunit)

	FGF7	NM_002009	4.1	Fibroblast growth factor 7 (keratinocyte growth factor)

	ELN	AA479278	2.5	Elastin (supravalvular aortic stenosis, Williams-Beuren syndrome)

	IGFBP7	NM_001553	2.4	Insulin-like growth factor binding protein 7

	SKAP2	AB014486	2.2	Src kinase associated phosphoprotein 2

	ARNT	AI768497	2.3	Aryl hydrocarbon receptor nuclear translocator

	FOXO1	NM_002015	1.5	Forkhead box O1

	BCAT1	AL390172	1.5	Branched chain aminotransferase 1, cytosolic

	ACVR2A	AI149508	1.5	Activin A receptor, type IIA

	TCF7L2	AA664011	1.4	Transcription factor 7-like 2 (T-cell specific, HMG-box)

	CHRNB2	NM_000748	1.4	Cholinergic receptor, nicotinic, beta 2 (neuronal)

	TCFL5	NM_006602	1.2	Transcription factor-like 5 (basic helix-loop-helix)

	NRP1	AA609131	-1.3	Neuropilin 1

	PHB	AL560017	-1.3	Prohibitin

	TBX5	NM_000192	-1.3	T-box 5

	NCK2	BC000103	-1.2	NCK adaptor protein 2

	PRPF19	NM_014502	-1.2	PRP19/PSO4 pre-mRNA processing factor 19 homolog (S. cerevisiae)

Integrin-mediated signaling pathway				

	ITGB2	NM_000211	6.6	Integrin, beta 2

	ITGB8	BF513121	3.6	Integrin, beta 8

	ITGA11	AF109681	1.5	integrin, alpha 11

	GAB2	NM_012296	1.5	GRB2-associated binding protein 2

Skeletal system/Tissue development				

	DSP	NM_004415	5.2	Desmoplakin

	FGF7	NM_002009	4.2	Fibroblast growth factor 7 (keratinocyte growth factor)

	SRGN	NM_002727	2.8	Serglycin

	INHBA	M13436	1.9	Inhibin, beta A

	TIMP3	AW338933	1.7	TIMP metallopeptidase inhibitor 3

	ENPP1	BF057080	1.7	Ectonucleotide pyrophosphatase/phosphodiesterase 1

	COL5A1	N30339	1.7	Collagen, type V, alpha 1

	ACVR2A	AI149508	1.5	Activin A receptor, type IIA

	FBN1	AW955612	1.4	Fibrillin 1

	BARX1	NM_021570N	-1.9	BARX homeobox 1

	GABBR1	NM_001470	-1.9	Gamma-aminobutyric acid (GABA) B receptor, 1

	HOXA11	H94842	-1.5	Homeobox A11

	CHST11	NM_018413	-1.5	Carbohydrate (chondroitin 4) sulfotransferase 11

	NSDHL	BC000245	-1.4	NAD(P) dependent steroid dehydrogenase-like

	ZNRD1	AF230337	-1.3	Zinc ribbon domain containing 1

	SMURF1	AF199364	-1.3	SMAD specific E3 ubiquitin protein ligase 1

DNA repair				

	ZSWIM7	BE645222	-1.6	Zinc finger, SWIM-type containing 7

	WDR33	AB044749	-1.5	WD repeat domain 33

	ASF1A	NM_014034	-1.5	ASF1 anti-silencing function 1 homolog A (S. cerevisiae)

	ATM	U82828	-1.3	ataxia telangiectasia mutated

	PMS2L1	AI375694	-1.3	Postmeiotic segregation increased 2-like 1

	ALKBH2	AI865555	-1.2	AlkB, alkylation repair homolog 2 (E. coli)

	XRCC6	NM_001469	-1.2	X-ray repair complementing defective repair in Chinese hamster cells 6

	DCLRE1A	D42045	-1.2	DNA cross-link repair 1A (PSO2 homolog, S. cerevisiae)

	PRPF19	NM_014502	-1.2	PRP19/PSO4 pre-mRNA processing factor 19 homolog (S. cerevisiae)

	ELN	AA479278	2.5	Elastin (supravalvular aortic stenosis, Williams-Beuren syndrome)

Cellular biosynthetic process				

	PITX2	NM_000325	-6.4	Paired-like homeodomain 2

	NR3C2	NM_000901	-5.8	Nuclear receptor subfamily 3, group C, member 2

	GALNT6	AW014155	-4.5	Polypeptide N-acetylgalactosaminyltransferase 6

	RGMB	BE855765	-2.7	RGM domain family, member B

	FOXF2	NM_001452	-2.2	Forkhead box F2

	ZNF529	AL109722	-2.1	Zinc finger protein 529

	BARX1	NM_021570	-1.9	BARX homeobox 1

	DHODH	M94065	-1.9	Dihydroorotate dehydrogenase

	LMO4	BC003600	-1.8	LIM domain only 4

	BRWD1	NM_018963	-1.7	Bromodomain and WD repeat domain containing 1

	TARS2	NM_025150	-1.7	Threonyl-tRNA synthetase 2, mitochondrial (putative)

	HLX	M60721	-1.7	H2.0-like homeobox

	BNC2	AI767962	-1.7	Basonuclin 2

	PGD	NM_002631	-1.6	Phosphogluconate dehydrogenase

	MEIS1	AL832770	-1.6	Meis homeobox 1

	ZNF420	AI339586	-1.6	Zinc finger protein 420

	ZFP28	AA831323	-1.5	Zinc finger protein 28 homolog (mouse)

	CARS2	NM_024537	-1.5	Cysteinyl-tRNA synthetase 2, mitochondrial (putative)

	HOXA11	H94842	-1.5	Homeobox A11

	PDLIM1	BC000915	13.2	PDZ and LIM domain 1 (elfin)

	ITGB2	NM_000211	6.6	Integrin, beta 2 (complement component 3 receptor 3 and 4 subunit)

	LMCD1	NM_014583	3.5	LIM and cysteine-rich domains 1

	KRT7	BC002700	3.1	Keratin 7

	ARNT	AI768497	2.3	Aryl hydrocarbon receptor nuclear translocator

	ZNF415	NM_018355	2.2	Zinc finger protein 415

	ZNF630	AK000580	2.0	Zinc finger protein 630

	INHBA	M13436	1.9	Inhibin, beta A

	GALNT1	U41514	1.7	Polypeptide N-acetylgalactosaminyltransferase 1

	ENPP1	BF057080	1.7	Ectonucleotide pyrophosphatase/phosphodiesterase 1

Miscellaneous				

	PTGES	AF010316	3.2	Prostaglandin E synthase

	ADAMTS5	NM_007038	1.7	ADAM metallopeptidase with thrombospondin type 1 motif, 5 (aggrecanase-2)

	DSP	NM_004415	5.2	Desmoplakin

	FMOD	NM_002023	2.7	Fibromodulin

	sFRP4	AW089415	3.4	Secreted frizzled-related protein 4

	MCAM	M28882	2.3	Melanoma cell adhesion molecule

	TNXB	NM_004381	-1.3	Tenascin XB

	FUS	NM_004960	-1.5	Fusion (involved in t(12;16) in malignant liposarcoma)

	TBX5	NM_000192	-1.3	T-box 5

*Positive number indicates elevated expression (fold) in OA meniscal cells compared to normal meniscal cells. Negative number indicates decreased expression (fold) in OA meniscal cells compared to normal meniscal cells.

We were particularly interested in identifying distinct differential gene expression patterns. As shown in Table 3, distinct differential gene expression patterns were apparent. For example, of the seven differentially-expressed genes classified in the immune response biological process, five genes had elevated expressions but only two genes had decreased expression in OA meniscal cells compared to the control meniscal cells. Of the three differentially-expressed genes classified in the inflammation response biological process, cytokine production biological process, calcium ion transport biological process or the biomineral formation biological process, all these genes had elevated expression in OA meniscal cells compared to the control meniscal cells. Of the four differentially-expressed genes classified in the regulation of phosphate metabolic process, three genes had elevated expression but only one gene had decreased expression in OA meniscal cells compared to the control meniscal cells. Of the eighteen genes classified in the cell proliferation biological process, thirteen genes had elevated expression but only five genes had decreased expression in OA meniscal cells compared to the control meniscal cells. Similar distinct expression patterns were also found in other categories of biological processes, including integrin-mediated signaling pathway and skeletal system/tissue development biological processes (Table 3).

In contrast, most of the genes with decreased expressions in OA meniscal cells compared to the control meniscal cells fell into a different set of biological processes. For example, of the ten differentially-expressed genes classified in the DNA repair biological process, nine genes had decreased expression but only one gene had elevated expression in OA meniscal cells compare to the control meniscal cells. Of the ninety-one differentially expressed genes classified in the cellular biosynthetic process biological process, seventy-four genes had decreased expression in OA meniscal cells and only seventeen genes had elevated expression (Partial results are listed in Table 3). These distinct distributions of the differentially-expressed genes in different sets of biological processes suggest that OA meniscal cells are different from normal meniscal cells. We also analyzed the differential gene expression between individual OA meniscal cells (OA1, OA2, OA3, OA4, and OA5) and the normal control meniscal cells together as a control group. Selected results are listed in Table 4.

**Table 4:** Genes differentially expressed in individual OA meniscal cells compared to normal meniscal cells.

Gene Name	Gene ID	OA1	OA1	OA3	OA4	OA5	Description
BST1	NM_004334	3.9	3.4	3.3	1.6	2.2	bone marrow stromal cell antigen 1

FGF9	NM_002010	3.7	2.4	3.7	2.5	2.9	Fibroblast growth factor 9

ACAN	NM_013227	2.4	1.6	1.6	0.0	1.6	Aggrecan

COL11A1	J04177	0.0	11.5	5.8	2.2	3.6	Collagen, type XI, alpha 1

ANKH	AL833238	0.0	1.8	1.7	1.5	1.8	Ankylosis, progressive homolog (mouse)

MGP	NM_000900	11.8	4.7	21.6	0.0	17.6	Matrix Gla protein

TUFT1	NM_020127	2.8	2.7	1.9	0.0	0.0	Tuftelin 1

TFIP11	NM_012143	2.2	1.6	0.0	0.0	0.0	Tuftelin interacting protein 11

IL20RB	AL578102	2.3	2.7	0.0	2.6	7.8	Interleukin 20 receptor beta

IL26	NM_018402	1.7	1.6	1.6	0.0	3.0	Interleukin 26

COL4A5	AW052179	-3.1	-3.0	-3.1	-3.0	-3.1	Collagen, type IV, alpha 5 (Alport syndrome)

MMP9	NM_004994	-2.2	-2.1	-2.1	0.0	-2.2	Matrix metallopeptidase 9

MMP10	NM_002425	-2.7	-2.6	-2.7	-1.5	-2.6	Matrix metallopeptidase 10

MMP12	NM_002426	-2.1	-2.0	-2.1	0.0	-2.1	Matrix metallopeptidase 12

FZD10	NM_007197	-1.6	-1.5	-1.6	-1.5	-1.5	Frizzled homolog 10 (Drosophila)

CYTL1	NM_018659	-2.6	-2.6	-2.6	-2.6	-2.5	Cytokine-like 1

CHODL	NM_024944	-2.6	-2.6	-2.6		-2.6	Chondrolectin

*Positive number indicates elevated expression (fold) in OA meniscal cells compared to normal meniscal cells. Negative number indicates decreased expression (fold) in OA meniscal cells compared to normal meniscal cells. 0.0 indicates there was no differential gene expression being detected.

### Validation of differential expression of selected genes

The genes selected for validation by quantitative real-time PCR included seven genes that displayed elevated expression in OA meniscal cells compared to normal meniscal cells (Table 5). They were major histocompatibility complex, class II, DP alpha (*HLA-DPA1*), integrin, beta 2 (*ITGB2*), ectonucleotide phosphodiesterase 1 (*ENPP1*), ADAM metallopeptidase with thrombospondin type 1 motif 5 (*ADAMTS5*), prostaglandin E synthase (*PTGES*), etc. Matrix metalloproteinase 10 (*MMP10*), which displayed decreased expression in OA meniscal cells compared to normal meniscal cells, was also selected for validation. RNA sample extracted from OA1 meniscal cells (Table 4) and RNA sample (mixture) extracted from the three normal control meniscal cells were used in the quantitative real time RT-PCR experiments. As shown in Table 5, the differential expressions of these genes were confirmed.

**Table 5:** Differential gene expression confirmed by real time RT-PCR*

Gene name	Gene ID	Differential Expression* Microarray	Differential Expression* RT-PCR	Description
HLA-DPA1	M27487	5.3	10.5	Major histocompatibility complex, class II, DP alpha

ITGB2	NM_000211	6.6	5.2	Integrin, beta 2

PTGES	AF010316	3.2	3.4	Prostaglandin E synthase

ENPP1	BF057080	1.7	2.1	Ectonucleotide pyrophosphatase/phosphodiesterase 1

ADAMTS5	BI254089	1.7	1.9	ADAM metallopeptidase with thrombospondin type 1 motif, 5

IL26	NM_018402	1.7	4.3	Interleukin 26

TUFT1	NM_020127	2.8	3.9	Matrix metalloproteinase 1

MMP10	NM_002425	-2.7	-3.1	Matrix metalloproteinase 10

*Differential Expression -- the numbers are the ratio of the relative expression level of a specific gene in OA1 meniscal cells to the relative expression level of the specific gene in the control meniscal cells (mixture of RNA prepared from the three normal control meniscal cells).

## Discussion

In the present study, we found that more than eighty five percent (85.7%) of the OA patients who underwent joint replacement surgery in our Medical Center had severe degenerative menisci (grades 4 or 3), indicating that meniscal degeneration is common in OA patients. We also found that meniscal degeneration correlated positively with articular cartilage degeneration in OA patients. The Spearman's correlation coefficient (r) was 0.672 (p < 0.0001). Our finding is consistent with previous reports that meniscal degeneration/tears is a feature of OA knee joints [7], that meniscal degeneration contributes to joint space narrowing [10], that there is a strong association between meniscal damage and cartilage loss [9], and that degenerative meniscal tears are positively associated with the severity of articular degeneration compared with other types of meniscal tears [14]. Taken together, our findings suggest that meniscal degeneration in OA, similar to cartilage degeneration, is a major degenerative process regardless of whether it is primary or secondary. Further studies such as examination of the complete patient histories may provide information regarding the cause-result relationship between meniscal degeneration and cartilage degeneration.

If meniscal degeneration is a general feature of OA, one would like to assess whether OA meniscal cells are different from normal meniscal cells and may play a role in the development of OA. To this end, we examined the differential gene expression between OA meniscal cells and normal control meniscal cells. We have recently reported that numerous genes that were classified in the biological process of immune response displayed elevated expression in OA FLS compared to rheumatoid arthritis (RA) FLS. *HLA-DPA1 *was expressed in OA FLS (hTERT-OA 13A FLS) 16 fold higher than that in RA FLS (hTERT-RA 516 FLS) [4]. Consistently, many genes that were classified in the biological process of immune response including *HLA-DPA1 *also displayed elevated expression in OA meniscal cells (Table 3). The findings indicate that OA meniscal cells, similar to OA FLS, may be involved in the inflammatory process observed in OA.

Calcium-containing crystals are found in the joint fluid of up to 65% of OA patients, and the presence of these crystals correlates with the radiographic evidence of cartilaginous degeneration [15-18]. There is evidence indicating that crystals may promote joint degeneration [19-21]. Most recently, it was demonstrated that the inhibition of meniscal calcification by calcium phosphocitrate, a potent anti-calcification agent, was accompanied by a significant reduction in the degeneration of articular cartilage in Hartley guinea pigs [22]. These data suggest that excessive meniscal calcification may play a role in OA. However, there have been no studies to investigate the alterations in OA meniscal cells or to identify the candidate disease genes that are potentially responsible for the excessive meniscal calcification in OA. In the present study, we found that many genes which are involved in biomineral formation such as *ENPP1 *[23], in phosphate metabolic process such as *ITGB2 *and in calcium ion transport such as calcium channel, voltage-dependent, L type, alpha 1C subunit (*CACNA1C*) are expressed at an elevated levels in OA meniscal cells compared to normal meniscal cells. These findings are consistent with clinical observations that meniscal calcification is more severe in OA menisci [24,25] and that calcium content in OA menisci is positively correlated with the stage of meniscal degeneration [26]. In addition to the genes listed in Table 3, several other genes that have been previously implicated in pathological calcification were also detected (Table 4). They were ankylosis progressive homolog (*ANKH*) [27,28], matrix Gla protein (*MGP*) [29,30] and tuftelin (*TUFT1*) [31-33]. Taken together, our findings suggest that OA meniscal cells may be actively involved in the meniscal calcification process in OA.

Many genes that have been previously found to be expressed at elevated levels in other types of OA cells or tissues such as in OA articular cartilage and OA bone were also detected in this study. They were integrin, beta 8 (*ITGB8*) [34], insulin-like growth factor binding protein 7 (*IGFBP7*) [34], fibromodul (*FMOD*) [28], cathepsin S (*CTSS*) [35], secreted frizzled-related protein 4 (*SFRP4*) [34], bone marrow stromal cell antigen 1 (*BST1*) [35], collagen, type XI, alpha 1 (*COL11A1*) [34], collagen, type V, alpha 1 (*COL5A1*) [34], *PTGES *[36] and *ADAMTS5 *[37]. These genes were expressed at significantly elevated levels in OA meniscal cells compared to normal meniscal cells (Tables 3 and 4). ADAMTS5 is a major cartilage matrix degrading enzyme and has been implicated in articular cartilage degeneration previously [38,39]. The elevated expression of ADAMTS5 in OA meniscal cells suggests that ADAMTS5 may play a role in meniscal degeneration.

Furthermore, many genes that have been previously found to be expressed at decreased levels in other types of OA cells or tissues were also detected in this study. They are *MMP10 *[34], *MMP12 *[40], tenascin XB (*TNXB*) [34], fusion (*FUS*) [28], melanoma cell adhesion molecule (*MCAM*) [35], and T-box 5 (*TBX5*) [5]. These genes were expressed at decreased levels in OA meniscal cells compared to normal meniscal cells (Tables 3 and 4). The consistency between our findings and the previous findings provides strong support for our hypothesis that OA meniscal cells are different from normal meniscal cells and may play an active role in the development of OA.

Our study has some limitations which should be considered. The control meniscal cells we used were derived from the menisci of osteosarcoma patients and were not optimal normal control meniscal cells. To minimize this limitation, we only collected overtly normal-appearing meniscal and cartilage specimens (grade 0) from osteosarcoma patients whose tumors were located far away from the knee joints. In addition, we analyzed the differential gene expression using meniscal cells rather than using meniscal tissue specimens directly to eliminate the effect of different drugs that might be taken by OA and osteosarcoma patients at the time of surgery.

Most meniscal cells synthesize type I collagen as their major collagen product. The meniscal cells in the inner, central nonvascularized region of meniscus synthesize type II collagen. It is an open question at this time as to whether a single cell type exists in the meniscus that displays a fibroblast-like phenotype or a chondrocyte-like phenotype depending on its environment, or whether two or more distinct cell types exist. Therefore, it is not absolutely certain at this time whether the differences in the gene expressions detected in this study reflect the overall differences between OA meniscal cells and the control meniscal cells or only reflect the differences between a subpopulation of OA meniscal cells and a subpopulation of the control meniscal cells. Another limitation is that the age of osteosarcoma patients was younger than the age of OA patients. Therefore, certain genes we detected may be age-related rather disease-specific. It is difficult to obtain age-matched control meniscal specimens because osteosarcoma occurs often in younger patients while OA occurs mostly in older patients. We will continue this line of study when more age matched normal control meniscal specimens become available in the future. In spite of these limitations, the consistency between our findings and the previous findings that many of the genes we detected are also abnormally expressed in other OA cell types/tissues [4,5,23,28,34-36,40] suggests that many of the differential gene expressions detected in this study are disease-specific.

## Conclusions

Our findings suggest that OA is not merely a cartilage disease, but also a disease of the menisci. OA meniscal cells may play an active role in the disease process of OA. Investigation of the gene expression profile of OA meniscal cells compared to normal meniscal cells may reveal new therapeutic targets for OA therapy and uncover novel disease markers for early diagnosis of OA.

## Abbreviations

OA: osteoarthritis; FLS: fibroblast-like synoviocytes; MMP: matrix metalloproteinase; ITGB: integrin: beta; ADAMTS5: metallopeptidase with thrombospondin type 1 motif 5; ANKH: ankylosis: progressive homolog; BST1: bone marrow stromal cell antigen 1; CACNA1C: calcium channel: voltage-dependent: L type: alpha 1C subunit; COL5A1: collagen, type V, alpha 1; COL11A1: collagen, type XI, alpha 1; CTSS: cathepsin S; ENPP1: ectonucleotide pyrophosphatase/phosphodiesterase 1; FGF7: fibroblast growth factor 7; FMOD: fibromodul; FUS: fusion; GAPDH: glyceraldehyde-3-phosphate dehydrogenase; HLA-DPA1: major histocompatibility complex, class II, DP alpha 1; IGFBP7: insulin-like growth factor binding protein 7; MCAM: melanoma cell adhesion molecule; MGP: matrix Gla protein; PTGES: prostaglandin E synthase; SFRP4: secreted frizzled-related protein 4; TUFT1: tuftelin; TBX5: T-box 5; TNXB: tenascin XB.

## Competing interests

The authors declare that they have no competing interests.

## Authors' contributions

YS, HEG and ENH conceived the study and participated in design and coordination. YS wrote the manuscript. DRM and JSK provided surgical tissues and participated in the discussion of experimental results. HJN assisted with statistical analysis. PRH, DRM and YS graded menisci and articular cartilage. PRH prepared cell culture and extracted RNA. HEG assisted with manuscript preparation. All authors read and approved the final manuscript.

## Pre-publication history

The pre-publication history for this paper can be accessed here:

http://www.biomedcentral.com/1471-2474/11/19/prepub
